# Supplementary figures and images for: Proximity biotinylation reveals novel secreted dense granule proteins of Toxoplasma gondii bradyzoites
Source: PLoS One. 2020 May 6;15(5):e0232552. doi: 10.1371/journal.pone.0232552 (PMC7202600; doi:10.1371/journal.pone.0232552)

# Figure 1

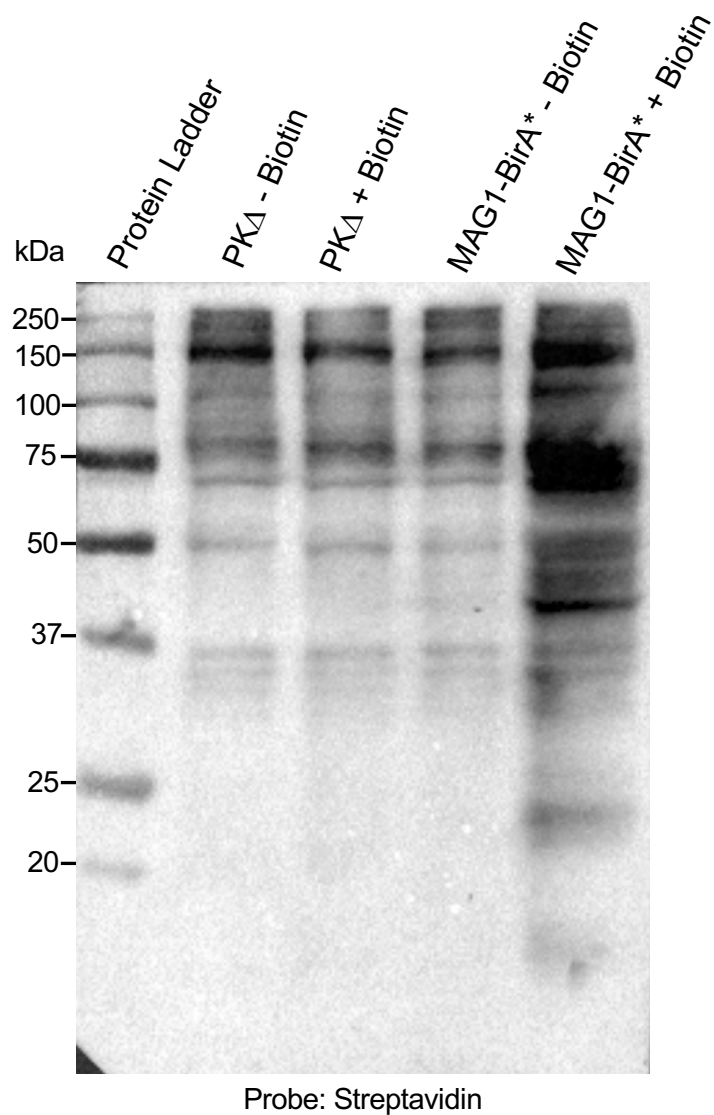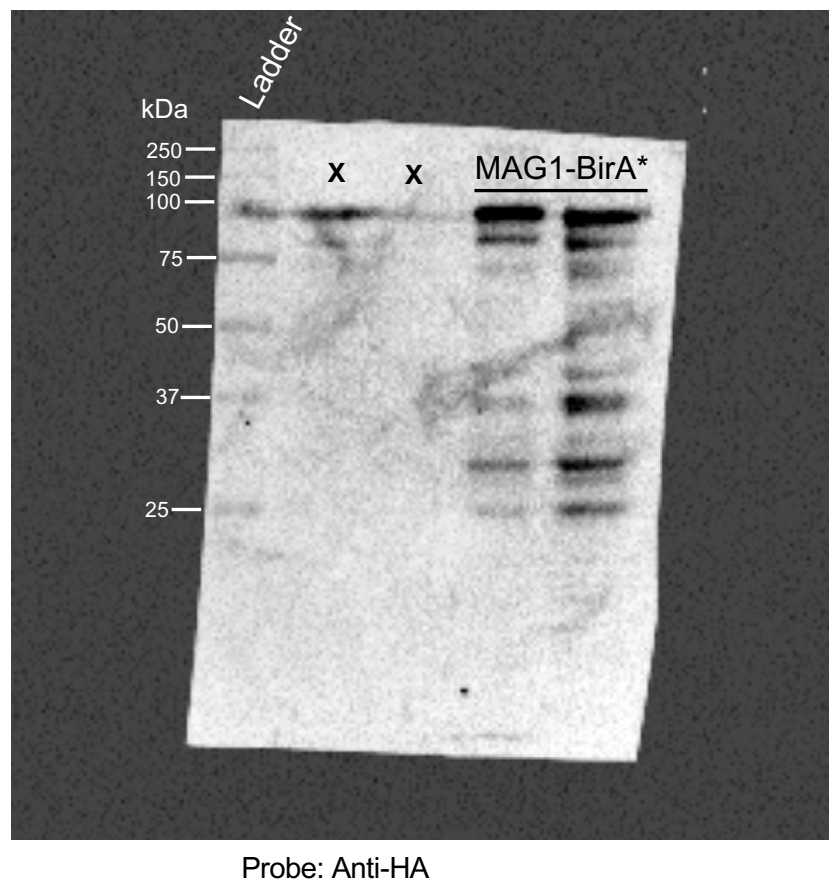

# Figure 3

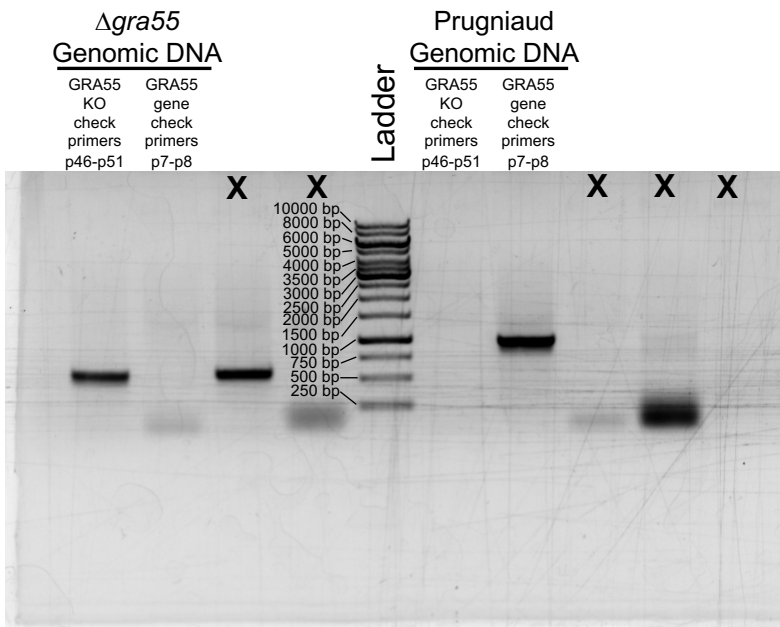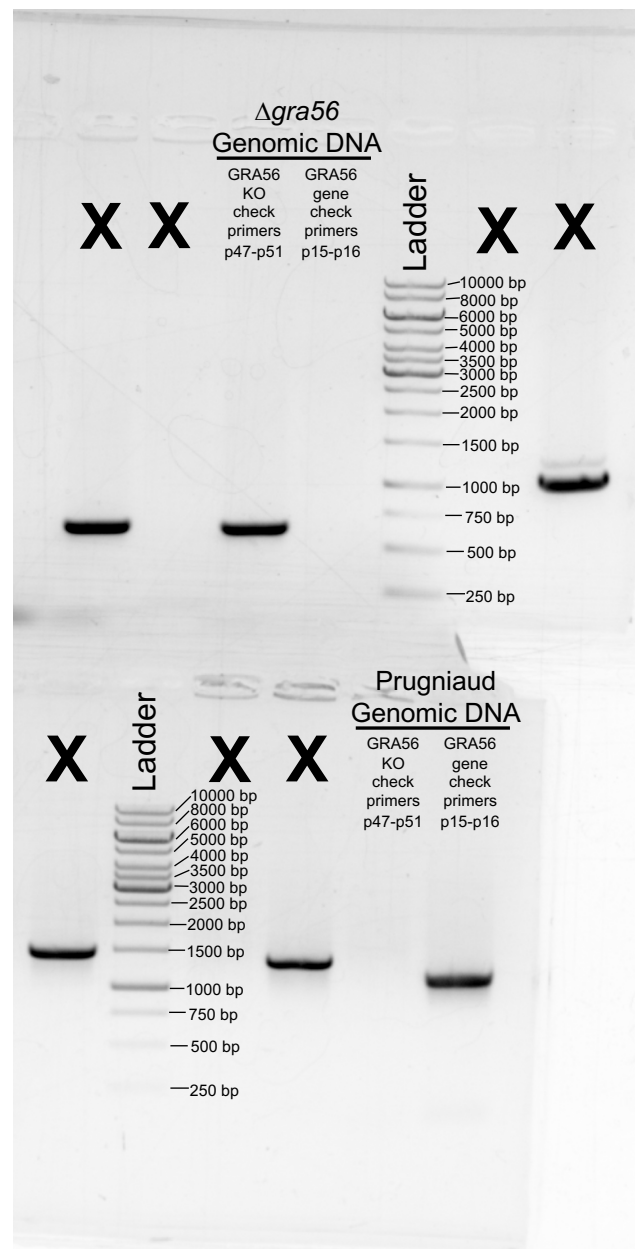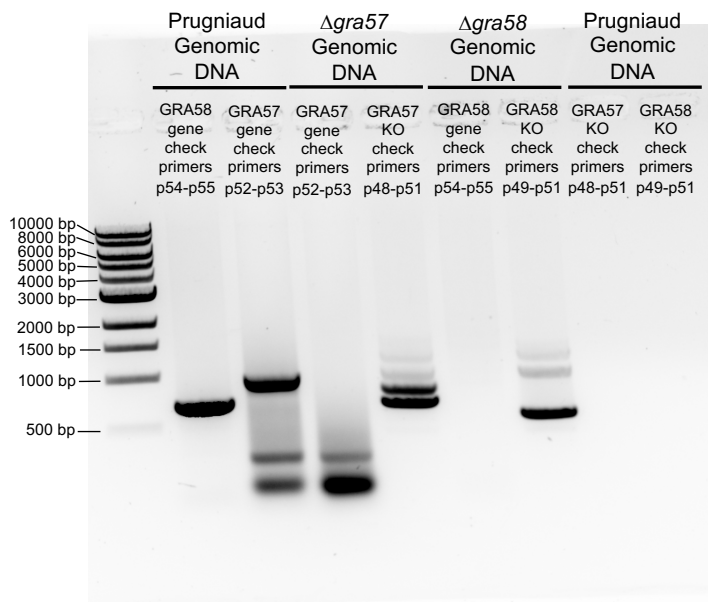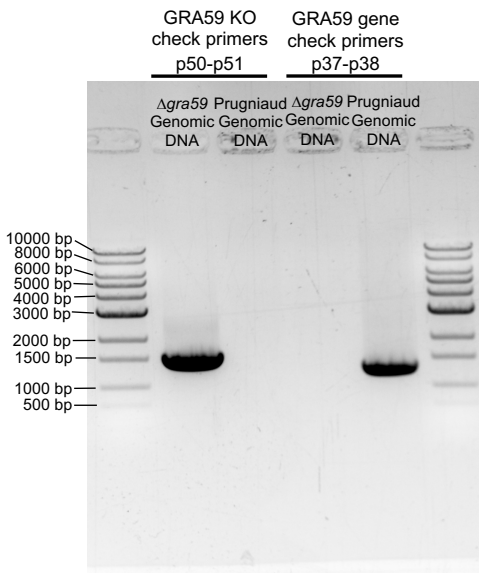

# Figure 4

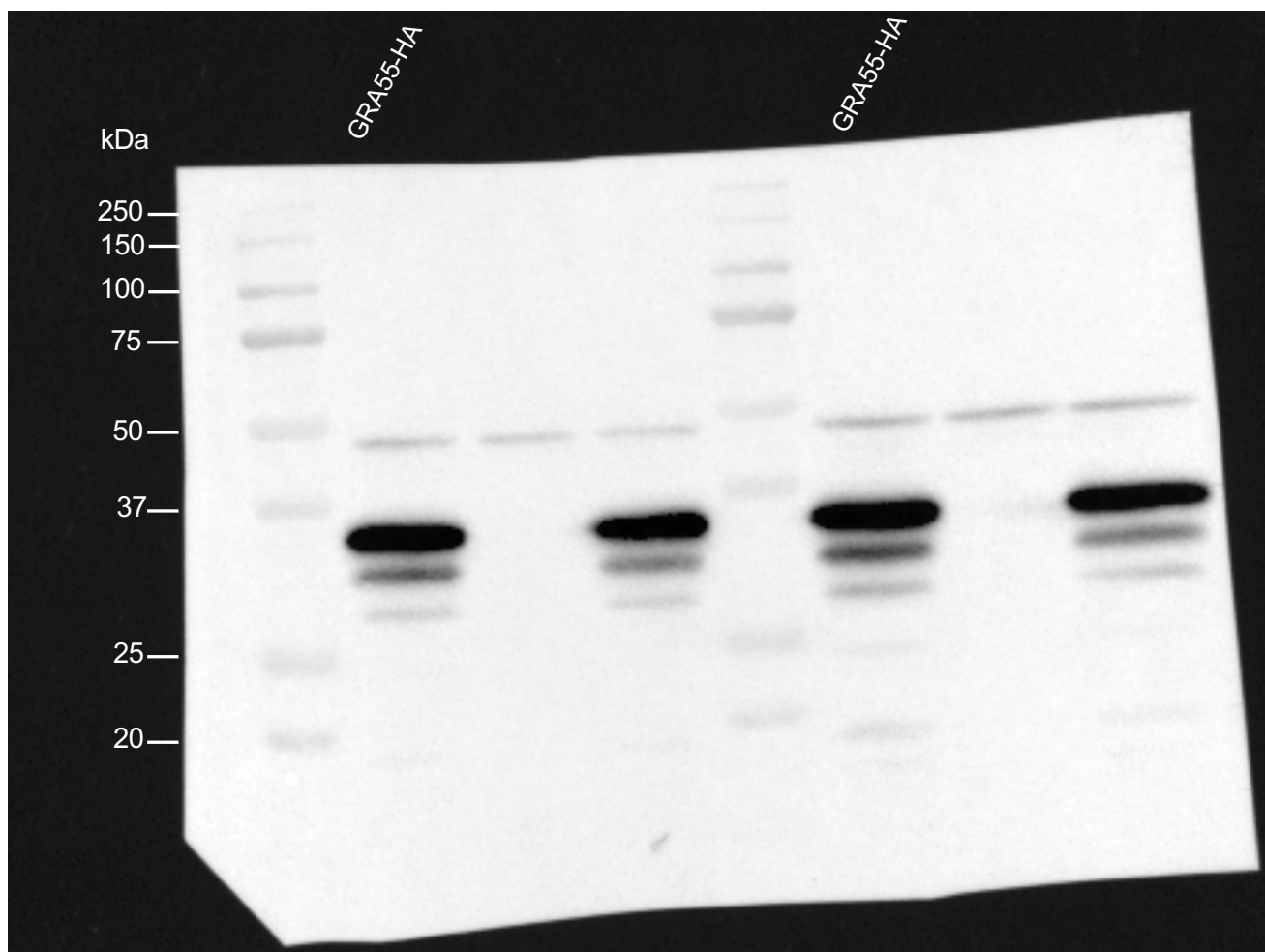

Supplement: S1 Raw images — (PDF) [file pone.0232552.s001.pdf]

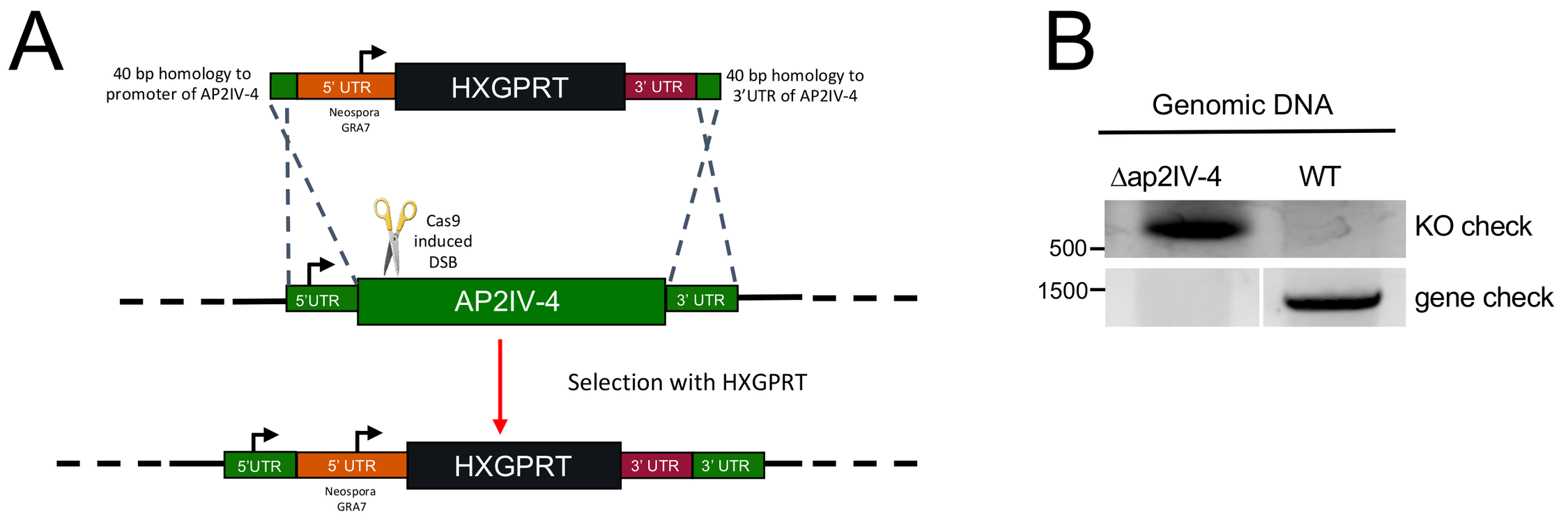

Supplement: S1 Fig — (A) Diagram of the construct used to delete AP2IV-4. Cas9 induced double stranded break (DSB) is represented by the scissors. The AP2IV-4 locus was replaced with an HXGPRT selectable marker using homologous recombination simultaneously with a Cas9 induced DSB. (B) PCR verification of deletion of AP2IV-4 and confirmation of homologous recombination of the HXGPRT cassette in place of the deleted locus. (TIF) [file pone.0232552.s002.tif]

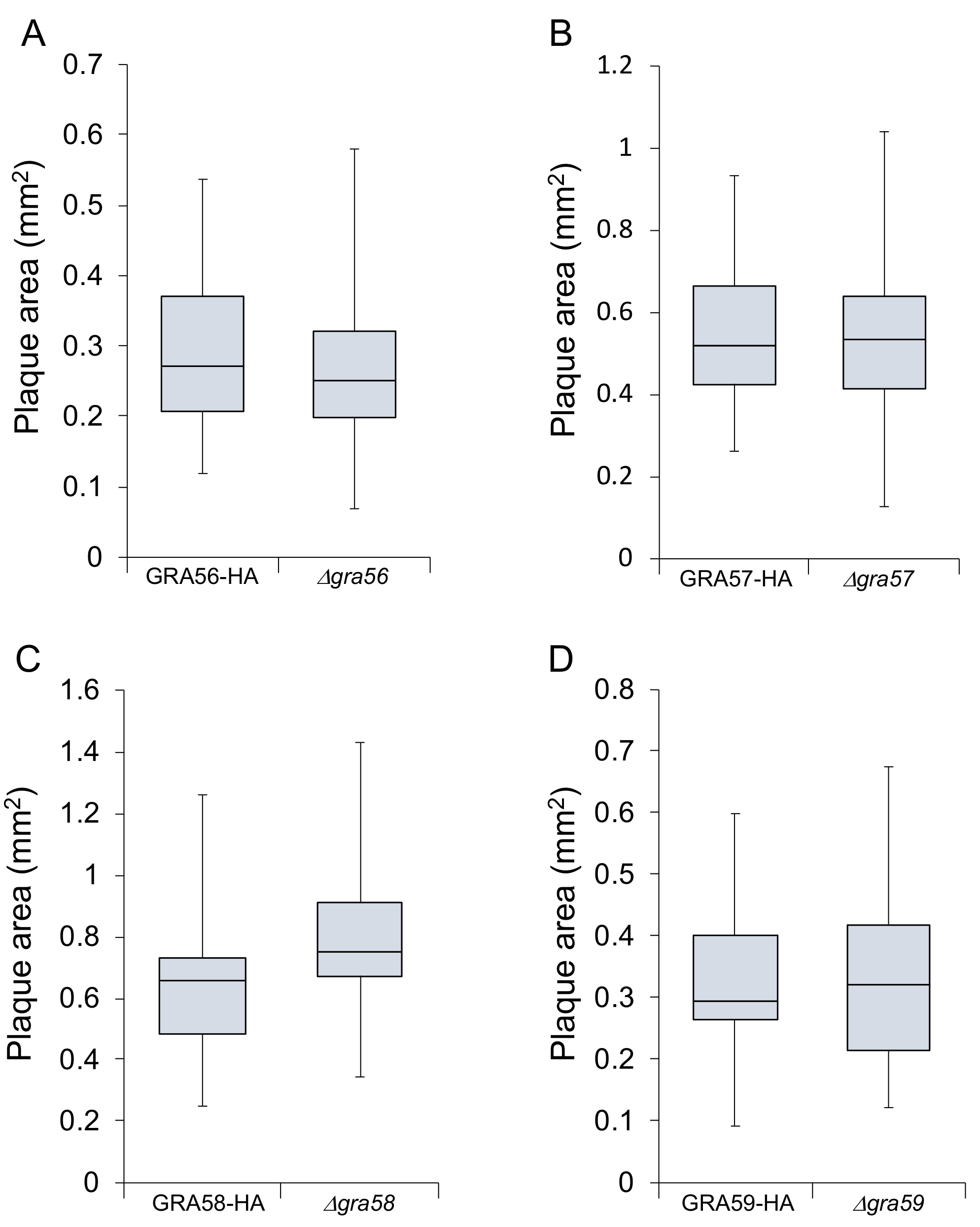

Supplement: S2 Fig — (A) GRA56, (B) GRA57, (C) GRA58, (D) GRA59. Results are shown as box-whisker plot with the middle line representing the median, the bottom and top of box representing the 25th and 75th percentile, and whiskers corresponding to smallest and largest plaques. (TIF) [file pone.0232552.s003.tif]

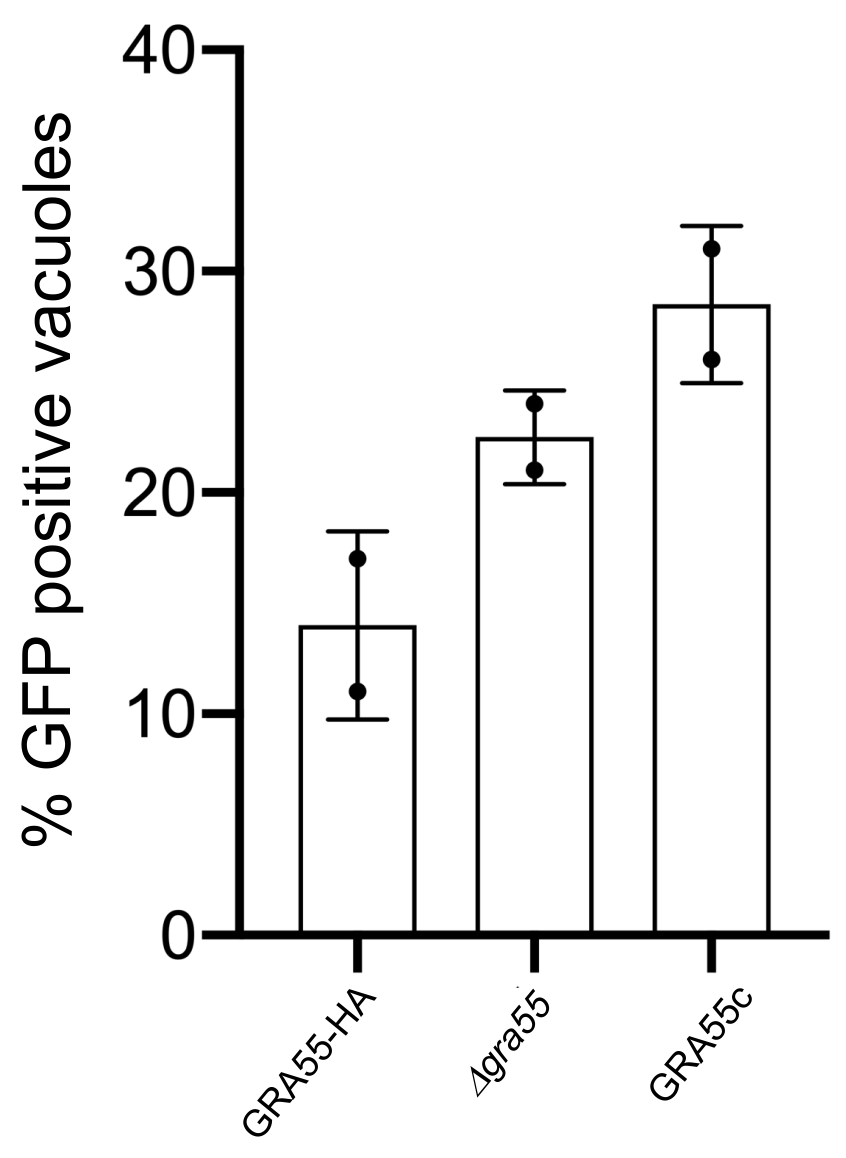

Supplement: S3 Fig — The mean ±SD was plotted for n = 2 biological replicates, where percentage of GFP positive vacuoles were calculated for each strain. No significance (p < 0.05) was reported comparing the means of GRA55-HA with Δgra55 or GRA55c with Δgra55 using a one-way ANOVA test. (TIF) [file pone.0232552.s004.tif]
