# Supplementary material for: Proximity biotinylation reveals novel secreted dense granule proteins of Toxoplasma gondii bradyzoites
Source: PLoS One. 2020 May 6;15(5):e0232552. doi: 10.1371/journal.pone.0232552 (PMC7202600; doi:10.1371/journal.pone.0232552)
Supplement: S2 Table — (DOCX) [file pone.0232552.s006.docx]

| **Supplemental Table S2**. Oligonucletoides and Primers used in this study  (Ligation/Endonuclease sites underlined) | | |
| --- | --- | --- |
| **#** | **Description** | **Sequence** |
| p1 | AP2IV-4 KO sgRNA fwd | AAG TTG CCT ATC CAT CTT TTC CAG TG |
| p2 | AP2IV-4 KO sgRNA rev | AAA ACA CTG GAA AAG ATG GAT AGG CA |
| p3 | AP2IV-4 KO FLANK fwd | AGG CAG TGG CTT CCA CAG GCA GTC CTC ACG CCA CCC ACC CCA CTC CAT GGA ACC TGA CTG |
| p4 | AP2IV-4 KO FLANK rev | TAC ACG ATA TTT TGA AAA GGA AAT GCG TTC CTG CTG CCC CCT GCA AGT GCA TAG AAG GAA |
| p5 | MAG1 LIC fwd | TAC TTC CAA TCC AAT TTA GCC GTT GGT TGT TCT GTG TG |
| p6 | MAG1 LIC rev | TCC TCC ACT TCC AAT TTT AGC AGC TGC CTG TTC CGC TAA GAT |
| p7 | GRA55 (309760) LIC fwd | TAC TTC CAA TCC AAT TTA GGT CGG AAT CTC ACG TTA CGT A |
| p8 | GRA55 (309760) LIC rev | TCC TCC ACT TCC AAT TTT AGC GCC CGC GTT GTC ACT GGC |
| p9 | GRA55 KO gRNA fwd | AAG TTG TCG GAA TCT CAC GTT ACG TG |
| p10 | GRA55 KO gRNA rev | AAA ACA CGT AAC GTG AGA TTC CGA CA |
| p11 | GRA55 KO FLANK fwd | CTG AGG TGC CTC AGT GTT TGT GTC CGT CGT CCC AGA GAG CCA CTC CAT GGA ACC TGA CTG |
| p12 | GRA55 KO FLANK rev | CTT GAC AAT GCC CTC GAC TCG GTT GCA AGA GGT GCC CTT CCT GCA AGT GCA TAG AAG GAA |
| p13 | GRA55 Complement fwd | ACT AGT CCG CAT GCT TAG AAG TGT GC |
| p14 | GRA55 HA Complement rev | GTA CTT AAT TAA TTA GGC ATA ATC TGG AAC ATC GTA |
| p15 | GRA56 (309930) LIC fwd | TAC TTC CAA TCC AAT TTA GGA GTG TCG GTG TTA CGA AG |
| p16 | GRA56 (309930) LIC rev | TCC TCC ACT TCC AAT TTT AGC CAT CTC CCT GTC TAT CCG C |
| p17 | GRA56 KO gRNA fwd | AAG TTG GAC ACG TCG AAC AAG TCA GG |
| p18 | GRA56 KO gRNA rev | AAA ACC TGA CTT GTT CGA CGT GTC CA |
| p19 | GRA56 KO FLANK fwd | GTT TTC CCG TAC TTG CAC GGT TCC AAG GAT TAG CCA GCC CCA CTC CAT GGA ACC TGA CTG |
| p20 | GRA56 KO FLANK rev | TTA CCG TTC CTT CCC GGT TGT TAA ACC ATT CGT CGT CTC CCT GCA AGT GCA TAG AAG GAA |
| p21 | GRA57 (217680) TAG gRNA fwd | AAG TTG TTC GTG GAC TTG ATG TAT TG |
| p22 | GRA57 (217680) TAG gRNA rev | AAA ACA ATA CAT CAA GTC CAC GAA CA |
| p23 | GRA57 (217680) TAG FLANK fwd | GAC ACC CGA GTA CGA GGA GCT CGG GTT ATT TGA TGT TGT AGA AGT GGA GGA CGG GAA TTC |
| p24 | GRA57 (217680) TAG FLANK rev | CCG GCA TCG CCG CCT GCC TCT GCT ATT TCT GTT TAT CAC GAC GGC CAG TGA ATT GTA ATA |
| p25 | GRA57 KO gRNA fwd | AAG TTG GTG TGG GCC GTT GGA CAG AG |
| p26 | GRA57 KO gRNA rev | AAA ACT CTG TCC AAC GGC CCA CAC CA |
| p27 | GRA57 KO FLANK fwd | ACT AGT AGG GAC GGA GAC GCT CGG CCG TTG GCA GCT GTC CCA CTC CAT GGA ACC TGA CTG |
| p28 | GRA57 KO FLANK rev | CCG AAT ACA TCA AGT CCA CGA ACA GGA GCG TTT TCC CCG CCT GCA AGT GCA TAG AAG GAA |
| p29 | GRA58 (268790) TAG gRNA fwd | AAG TTG ATT GAC GCT CAA ACA ATG TG |
| p30 | GRA58 (268790) TAG gRNA rev | AAA ACA CAT TGT TTG AGC GTC AAT CA |
| p31 | GRA58 (268790) TAG FLANK fwd | CCC TCC CAG CGA CGA CAT TGT ACA GAG CAC CTC AGA TGT TGA AGT GGA GGA CGG GAA TTC |
| p32 | GRA58 (268790) TAG FLANK rev | TAT TGT TGT GTT CAT TGG CTC CAT TGG CAC ACA CGT GCC GAC GGC CAG TGA ATT GTA ATA |
| p33 | GRA58 KO gRNA fwd | AAG TTG GTT CGT GTC CTG CTC CAT AG |
| p34 | GRA58 KO gRNA rev | AAA ACT ATG GAG CAG GAC ACG AAC CA |
| p35 | GRA58 KO FLANK fwd | CAG ATG GCA AGC GCT ACT CTG TGG TGG TAC GGA GAA GGA CCA CTC CAT GGA ACC TGA CTG |
| p36 | GRA58 KO FLANK rev | CCG TAA GAC AGC ACG GTC AAC AAG GAA CGG GTT AAG TTA CCT GCA AGT GCA TAG AAG GAA |
| p37 | GRA59 (313440) LIC fwd | TAC TTC CAA TCC AAT TTA GCT GCC GAG CAC TCA TTC C |
| p38 | GRA59 (313440) LIC rev | TCC TCC ACT TCC AAT TTT AGC GTC ACG AGG GGT ACC CG |
| p39 | GRA59 KO gRNA fwd | AAG TTG TCG GTT AAG AGT ATC CGA GG |
| p40 | GRA59 KO gRNA rev | AAA ACC TCG GAT ACT CTT AAC CGA CA |
| p41 | GRA59 KO FLANK fwd | CAT AGT TAA AAA TAT ACT GCG AGA AGG AAA TGG CAA AAC CCA CTC CAT GGA ACC TGA CTG |
| p42 | GRA59 KO FLANK rev | CGC TAG ACG ATT AGG GAA GCG TTC TGC GAT CGC ACT CAG CCT GCA AGT GCA TAG AAG GAA |
| p43 | AP2IV-4 KO check fwd | CGT CTC ACA TCC ATC CTG CC |
| p44 | AP2 IV-4 gene check fwd | GCT CTC CTG CCT AAC ACA AC |
| p45 | AP2 IV-4 gene check rev | CCT TCA CGA CTT GTG CTG G |
| p46 | GRA55 KO check fwd | GTC CCG AAA CTT GAA GAA CAA TTG |
| p47 | GRA56 KO check fwd | CGA GTT CTC TTT GAA GAG GTG G |
| p48 | GRA57 KO check fwd | AAG ACC CAT TAC CAC AGT CG |
| p49 | GRA58 KO check fwd | ATG AAC CGC AGT CCC GAT AT |
| p50 | GRA59 KO check fwd | GCT GCA GAA AGC GAT GAC AG |
| p51 | NC_HPT KO check rev | GAA CTG CTT GCG AGA CCT G |
| p52 | GRA57 gene check fwd | AGT CTG TGT CAA ATC CGG GT |
| p53 | GRA57 gene check rev | GGT ACA TTT GCA TGT CGC CT |
| p54 | GRA58 gene check fwd | GAT GAT CCG TAT TTC TGG CC |
| p55 | GRA58 gene check rev | CTT GTC GAT TTC CGC CTC AA |
